# Historical Changes in Anesthesia Practice for Laparoscopic Gynecological Surgery at Juntendo University Hospital – A Retrospective Observational Study Focusing on Factors Affecting the Incidence of Postoperative Nausea and Vomiting

**DOI:** 10.14789/jmj.JMJ22-0013-OA

**Published:** 2022-10-15

**Authors:** OSAMU KUDOH, MASAKAZU HAYASHIDA

**Affiliations:** 1 Department of Anesthesiology and Pain Medicine, Juntendo University Faculty of Medicine, Tokyo, Japan; 1 Department of Anesthesiology and Pain Medicine, Juntendo University Faculty of Medicine, Tokyo, Japan

**Keywords:** atropine, droperidol, laparoscopic gynecological surgery, neostigmine, postoperative nausea and vomiting

## Abstract

**Objective:**

Previously, we reported that antiemetics (droperidol and/or dexamethasone) could significantly reduce the incidence of postoperative nausea and vomiting (PONV) after laparoscopic gynecological surgery (LGS). We retrospectively investigated anesthesia practice during the era earlier than the above-mentioned report to identify factors affecting PONV.

**Methods:**

We investigated 1,221 patients who underwent LGS at Juntendo University Hospital between 2007 and 2009. Effects of nine covariates likely to affect PONV on the actual incidence of PONV were examined with the multivariate logistic regression analysis.

**Results:**

The actual incidence of PONV developing until nine hours after the transfer to the ward was 47.3% (577/1,221) in the total cohort. The multivariate logistic regression analysis revealed that longer duration of anesthesia (in hours) was associated with the increased incidence of PONV (odds ratio [OR], 1.170; 95% confidence interval [CI], 1.000-1.360; *p* = 0.0467), the use of the reversal agent neostigmine co-administrated with atropine was associated with the lower incidence of PONV (OR, 0.746; 95% CI, 0.585-0.950; *p* = 0.0177), and no use of PCA and the use of fentanyl PCA without droperidol were associated with the higher incidence of PONV, compared with the use of fentanyl PCA with droperidol (OR, 1.810; 95% CI, 1.250-2.640; *p* = 0.0019; and OR, 2.500; 95% CI, 1.880-3.310; *p* < 0.0001; respectively).

**Conclusions:**

Longer duration of anesthesia was associated with the increased incidence of PONV. Addition of droperidol to the PCA infusate and the use of reversal agent neostigmine co-administrated with atropine were associated with the reduced incidence of PONV.

## Introduction

Postoperative nausea and vomiting (PONV) remain among the most common and annoying complications after general anesthesia^1)^. Especially, the incidence of PONV is extremely high after laparoscopic gynecological surgery (LGS) for benign diseases, affecting as many as 80% of patients^2)^. Such a high incidence has been attributed to multiple risk factors for PONV related to patients undergoing LGS for benign diseases, including female gender, types of surgery (laparoscopic, gynecological), nonsmoking status, younger age, and postoperative use of opioids^1)^. Although LGS is categorized as minimal invasive surgery due to small skin incisions, postoperative pain is not minimal after LGS due to the development of inflammatory visceral pain, and therefore, opioid analgesics, including those using intravenous patient-controlled analgesia (PCA), are often required for adequate pain control after LGS^3)^.

For improving patient satisfaction and enhancing patient recovery, antiemetic therapy to prevent PONV is essential^1)^. Previously, we reported that the incidence of PONV after LGS was 70% without an effective antiemetic prophylaxis, but the incidence could be reduced to 43% with a prophylactic antiemetic, either droperidol or dexamethasone, and further, to 17% with a combination of both drugs^4)^. After confirming these results, we incorporated the routine use of both drugs into our anesthesia practice for LGS. However, we have not investigated the usual anesthesia practice in LGS patients and the incidence of PONV before this change in the anesthesia protocol was made.

In the present study, we retrospectively investigated anesthesia practice in LGS patients during the era earlier than the above-mentioned protocol change to learn a history of anesthesia practice for LGS at our institute, and hopefully, to identify factors affecting PONV in patients undergoing LGS.

## Methods

This retrospective, observational study was approved by the Ethics Committee of Juntendo University Hospital (approval number, 15-173), with a waiver of written informed consent.

We investigated 1,236 consecutive patients who underwent LGS for benign diseases at Juntendo University Hospital between May, 2007 and January, 2009. We collected the following data from the anesthesia record: patients’ demographic characteristics, including age, body height, body weight, body mass index (BMI), and smoking habits; durations of anesthesia and surgery; surgical procedures performed; methods of general anesthesia, including general anesthetics, opioid analgesics, muscle relaxants, reversal agents for muscle relaxants, and prophylactic antiemetic agents given during anesthesia; the use or no use of intravenous fentanyl PCA for postoperative pain control, and contents of the PCA infusate, if used. Further, we collected the following data from the medical record: postoperative rescue analgesics, including opioid or non-opioid analgesics given in the post-anesthesia care unit (PACU) and/or ward, severity of PONV evaluated repeatedly on the four-category verbal rating scale (VRS; none, mild, moderate, and severe) in the PACU, severity of PONV evaluated on the 11-point numerical rating scale (NRS; 0 = none, 10 = worst imaginable) at 0, 3, 6, and 9 hours after the return to the gynecological ward, and rescue antiemetics given in the PACU and/or ward.

The use or no use of intravenous fentanyl PCA was left to the discretion of attending anesthesiologists. If PCA was used, postoperative analgesia was managed basically with intravenous PCA using a disposable infusion pump (Baxter Infusor, BB30LV4, Baxter Limited, Tokyo, Japan), filled with fentanyl 1000 μg and normal saline in a total volume of 80 ml, allowing for a constant infusion rate, 4 ml/h (fentanyl, 50 μg/h) and a bolus dose on demand, 2 ml (fentanyl 25 μg), with a 30-minute lockout time. Droperidol 2.5 or 5 mg was added or not added to the PCA infusate at the discretion of attending anesthesiologists.

Patients who continued to report ‘none’ on the nausea VRS in the PACU and ‘0’ on the nausea NRS in the ward, without developing vomiting nor receiving rescue antiemetics during the observation period until nine hours after the return to the gynecological ward were deemed to have no PONV. Otherwise, patients were deemed to have PONV.

## Statistical analysis

Data are expressed as Mean ± standard deviation (SD), Median [Interquartile Range] or Number (%) according to data types. Data were compared between groups using the unpaired *t* test, Mann-Whitney *U* test, or chi-square test accordingly. Based on the recent guideline^1)^, effects of nine factors likely to affect the development of PONV, including age, BMI, smoking habits, duration of anesthesia, the method of general anesthesia (inhalation anesthesia versus propofol-based total intravenous anesthesia [TIVA]), the use of prophylactic antiemetics during anesthesia, the use of the reversal agent for muscle relaxants, the use of intravenous fentanyl PCA, and the use of postoperative rescue opioids other than PCA fentanyl, were examined with univariate and multivariate logistic regression analyses. A *p* value < 0.05 was considered statistically significant. Statistical analysis was performed with the EZR (Easy R), available from the Jichi Medical University Saitama Medical Center at https://www.jichi.ac.jp/saitama-sct/SaitamaHP.files/download.html.

## Results

Among 1,236 patients studied, 15 patients were excluded because they were transferred to the non-gynecological ward and data on NRS nausea scores were lacking. Therefore, data from 1,221 patients were analyzed. Patients’ demographic, anesthetic, and surgical characteristics, and drugs used perioperatively are summarized in Table 1.

**Table 1:** Patients' demographic, anesthetic, and surgical characteristics, and drugs used perioperatively in the total cohort, in patients developing postoperative nausea and vomiting (PONV) (PONV (+)), and in patients not developing PONV (PONV (-))

		Total (*n* = 1221) *n* = 1221	PONV (+) *n* = 577 (47.3%)	PONV (-) *n* = 644 (52.7%)	*p* value
Age (years)		36.9 ± 6.9	36.7 ± 6.8	37.0 ± 7.1	0.5720
Body height (cm)		159 [155 - 163)	159 [155 - 162.5]	159 [155 - 163]	0.1960
Body weight (kg)		53.1 ± 7.9	53.1 ± 7.9	53.1 ± 7.9	0.8140
Body mass index (kg/m^2^)		21.0 ± 2.9	21.0 ± 2.9	20.9 ± 2.8	0.4870
Smoking habits	Smoker	85 (7.0%)	35 (6.1%)	50 (7.8%)	0.2444
	Non-smoker	1,136 (93.0%)	542 (93.9%)	594 (92.2%)	
Duration of Anesthesia (hours)		2.30 ± 0.77	2.34 ± 0.76	2.26 ± 0.77	0.0241
Duration of Surgery (hours)		1.68 ± 0.74	1.71 ± 0.74	1.65 ± 0.75	0.1160
Surgical procedures performed	Laparoscopic myomectomy	755 (61.8%)	379 (65.7%)	376 (58.4%)	0.0010
	Laparoscopic cystectomy	372 (30.5%)	168 (29.1%)	204 (31.7%)	
	Laparoscopic ectopic pregnancy surgery	45 (3.7%)	10 (1.7%)	35 (5.4%)	
	Laparoscopic salpingo-oophorectomy	36 (2.9%)	12 (2.1%)	24 (3.7%)	
	Other laparoscopic surgeries	13 (1.1%)	8 (1.4%)	5 (0.8%)	
Methods of general anesthesia	Inhalation anesthesia	1,196 (98.0%)	565 (97.9%)	631 (98.0%)	0.9400
	Total intravenous anesthesia (TIVA)	25 (2.0%)	12 (2.1%)	13 (2.0%)	
Opioid analgesics given during anesthesia	Remifentanil + Fentanyl	754 (61.8%)	364 (63.1%)	390 (60.6%)	0.2594
	Fentanyl	336 (27.5%)	148 (25.6%)	188 (29.2%)	
	Remifentanil	111 (9.1%)	58 (10.1%)	53 (8.2%)	
	No use	20 (1.6%)	7 (1.2%)	13 (2%)	
Muscle relaxants given during anesthesia	Vecuronium	1,116 (91.4%)	541 (93.8%)	575 (89.3%)	0.1128
	Rocuronium	92 (7.5%)	32 (5.5%)	60 (9.3%)	
	Suxamethonium + Vecuronium	9 (0.7%)	3 (0.5%)	6 (0.9%)	
	Suxamethonium + Rocuronium	1 (0.1%)	0 (0.0%)	1 (0.2%)	
	Suxamethonium	1 (0.1%)	0 (0.0%)	1 (0.2%)	
	No use	2 (0.2%)	1 (0.2%)	1 (0.2%)	
Reversal agent for muscle relaxants	Neostigmine combined with atropine	494 (40.5%)	207 (35.9%)	287 (44.6%)	0.0020
	No use	727 (59.5%)	370 (64.1%)	357 (55.4%)	
Prophylactic antiemetics given during anesthesia	Metoclopramide	96 (7.8%)	34 (5.9%)	62 (9.6%)	0.0033
	Droperidol	40 (3.3%)	12 (2.1%)	28 (4.3%)	
	No use	1,085 (88.9%)	531 (92.0%)	554 (86.0%)	
Intravenous fentanyl patient-controlled analgesia (PCA)	PCA without droperidol	616 (50.5%)	348 (60.3%)	268 (41.6%)	< 0.0001
	PCA with droperidol	384 (31.4%)	124 (21.5%)	260 (40.4%)	
	No use of PCA	221 (18.1%)	105 (18.2%)	116 (18.0%)	
Rescue analgesics given after anesthesia	Non-opioid analgesics	271 (22.1%)	133 (23.1%)	138 (21.4%)	0.0365
	Opioid analgesics	208 (17.0%)	113 (19.6%)	95 (14.8%)	
	No use	702 (60.8%)	331 (57.4%)	411 (63.8%)	
Rescue antiemetics given after anesthesia	Metoclopramide	351 (28.7%)	351 (60.8%)	0 (0.0%)	< 0.0001
	Droperidol	23 (1.9%)	23 (4.0%)	0 (0.0%)	
	Metoclopramide + Droperidol	15 (1.2%)	15 (2.6%)	0 (0.0%)	
	No use	832 (68.2%)	188 (32.6%)	644 (100.0%)	

Data are expressed as Mean ± Standard Deviation, Median [Interquartile Range], or Number (%). Data were compared between groups using the unpaired *t* test, Mann-Whitney *U* test, or chi-square test accordingly.

The results of the univariate and multivariate logistic regression analyses to examine effects of above-mentioned nine covariates on the development of PONV are summarized in Table 2. The univariate logistic regression analysis revealed that age (including 31 patients aged ≥ 50 years), BMI, smoking habits (had by 85 patients), or the method of general anesthesia (TIVA in 25 patients versus inhalation anesthesia in 1,196 patients) was not associated with or did not tend to be associated with the incidence of PONV (*p* > 0.1 for each), while the use of prophylactic antiemetics given in 136 patients (metoclopramide in 96 patients and droperidol in 40 patients), the use of the reversal agent for muscle relaxants (neostigmine [2 mg] co-administrated with atropine [1 mg]) given in 494 patients, postoperative uses of rescue opioid analgesics other than PCA fentanyl given in 208 patients (pentazocine, fentanyl, buprenorphine, and pethidine in 99, 79, 16, and 14 patients, respectively), and intravenous fentanyl PCA, including no use of PCA in 221 patients, the use of fentanyl PCA without droperidol in 616 patients, and the use of fentanyl PCA with droperidol in 384 patients, were significantly associated with the incidence of PONV (*p* < 0.05 for each), and duration of anesthesia tended to be associated with the incidence of PONV (*p* = 0.0689). However, the multivariate logistic regression analysis employing these nine covariates revealed that only three covariates, including duration of anesthesia, the use of the reversal agent, and the use or no use of fentanyl PCA with or without droperidol were significantly associated with the incidence of PONV (*p* < 0.05 for each). A longer duration of anesthesia (in hours) was associated with the increased incidence of PONV (odds ratio [OR], 1.170; 95% confidence interval [CI], 1.000-1.360; *p* = 0.0467). The use of the reversal agent was associated with the lower incidence of PONV (OR, 0.746; 95% CI, 0.585-0.950; *p* = 0.0177). No use of PCA and the use of fentanyl PCA without droperidol were associated with the higher incidence of PONV, compared with the use of fentanyl PCA with droperidol (OR, 1.810; 95% CI, 1.250-2.640; *p* = 0.0019; and OR, 2.500; 95% CI, 1.880-3.310; *p* < 0.0001, respectively), and the use of fentanyl PCA without droperidol tended to be associated with the higher incidence of PONV, compared with no use of PCA (OR, 1.380; 95% CI, 0.988-1.920; *p* = 0.0592).

**Table 2:** Results of univariate and multivariate logistic regression analyses to identify factors affecting postoperative nausea and vomiting (PONV) that developed in 47.3% of patients (577/1221)

	Univariate logistic regression analysis			Multivariate logistic regression analysis		
	ORs	95% CIs	*p* value	ORs	95% CIs	*p* value
Age (years)	0.993	0.977-1.010	0.4310	0.990	0.973-1.010	0.2540
Body mass index (kg/m^2^)	1.010	0.975-1.050	0.4940	1.010	0.973-1.060	0.5000
Smoking habits	0.767	0.490-1.200	0.2460	0.730	0.460-1.160	0.1820
Duration of anesthesia (hours)	1.150	0.990-1.330	0.0689	1.170	1.000-1.360	0.0467
Methods of anesthesia	0.970	0.439-2.140	0.9400	0.854	0.373-1.950	0.7080
Prophylactic antiemetics	0.533	0.367-0.776	0.0010	0.737	0.492-1.100	0.1380
Reversal agent for muscle relaxants	0.696	0.553-0.876	< 0.0001	0.746	0.585-0.950	0.0177
Postoperative rescue opioid analgesics	1.410	1.040-1.900	0.0253	1.230	0.887-1.700	0.2150
PCA with droperidol	1			1		
No use of PCA	1.900	1.350-2.670	0.0002	1.810	1.250-2.640	0.0019
PCA without droperidol	2.720	2.090-3.550	< 0.0001	2.500	1.880-3.310	< 0.0001
No use of PCA	1			1		
PCA without droperidol	1.430	1.050-1.950	0.0218	1.380	0.988-1.920	0.0592
PCA with droperidol	0.527	0.375-0.740	0.0002	0.552	0.379-0.802	0.0019

CIs, confidence intervals; ORs, odds ratios; PCA, intravenous fentanyl patient-controlled analgesia Regarding PCA, ORs, 95% CIs, and *p* values relative to PCA with droperidol and relative to no use of PCA were calculated.

Rescue analgesics and rescue antiemetics used postoperatively and the incidence of PONV according to differences in the use of fentanyl PCA are shown in Table 3. In the total cohort, the actual incidence of PONV that developed until nine hours after the return to the gynecological ward was 47.3% (577/1221) (Tables 1 & 3). Regarding intravenous fentanyl PCA, the actual incidences of PONV were 32.3% (124/384) in patients receiving fentanyl PCA with droperidol, 47.5% (105/221) in patients not receiving PCA, and 56.5% (348/616) in patients receiving fentanyl PCA without droperidol (Table 3). The chi-square test revealed that the incidence of PONV was lower in patients receiving fentanyl PCA with droperidol than those not receiving PCA (*p* = 0.0005) and those receiving fentanyl PCA without droperidol (*p* < 0.0001), and also was lower in patients not receiving PCA than those receiving fentanyl PCA without droperidol (*p* = 0.0227), almost in good agreement with results of logistic regression analyses.

**Table 3:** Rescue analgesics and rescue antiemetics given postoperatively and the incidence of postoperative nausea and vomiting (PONV) in the toral cohort, in patients receiving intravenous fentanyl patient-controlled analgesia (PCA) without droperidol, in patients receiving PCA with droperidol, and in patients not receiving PCA

		Total cohort (*n* = 1221)	PCA without droperidol (*n* = 616)	PCA with droperidol (*n* = 384)	No use of PCA (*n* = 221)	*p* value
Rescue analgesics other than PCA	Non-opioid analgesics	271 (22.2%)	131 (21.3%)	62 (16.1%)	78 (35.3%)	< 0.0001
	Opioid analgesics	208 (17.0%)	88 (14.3%)	26 (6.8%)	94 (42.5%)	
	No use	742 (60.8%)	397 (64.4%)	296 (77.1%)	49 (22.2%)	
Rescue antiemetics	Metoclopramide	351 (28.7%)	230 (37.3%)	57 (14.8%)	64 (29.0%)	< 0.0001
	Droperidol	23 (1.9%)	18 (2.9%)	4 (4.0%)	1 (0.5%)	
	Metoclopramide + Droperidol	15 (1.2%)	11 (1.8%)	2 (0.5%)	2 (0.9%)	
	No use	832 (68.2%)	357 (58.0%)	321 (83.6%)	154 (69.7%)	
PONV	Yes (+)	577 (47.3%)	348 (56.5%)	124 (32.3%)	105 (47.5%)	< 0.0001
	No (-)	644 (52.7%)	268 (43.5%)	260 (67.6%)	116 (52.5%)	

Data are expressed as Number (%). Data were compared among three groups using the chi-square test.

Postoperative rescue opioids were used in 6.8% (26/384) of patients receiving fentanyl PCA with droperidol, 14.3% (88/616) of patients receiving fentanyl PCA without droperidol, and 42.5% (94/221) of patients not receiving PCA (Table 3). The chi-square test revealed that rescue opioids were used more frequently in patients not receiving PCA than those receiving fentanyl PCA without droperidol (*p* < 0.0001), and in patients receiving fentanyl PCA without droperidol than those receiving fentanyl PCA with droperidol (*p* = 0.0003).

The chi-square test revealed that actual incidence of PONV was significantly lower in patients receiving the reversal agent (41.9% [207/494]) than those not receiving this agent (50.9% [370/727]) (*p* = 0.0020) in agreement with results of logistic regression analyses.

## Discussion

In the present study, the multiple logistic regression analysis revealed that longer duration of anesthesia was associated with the higher incidence of PONV; the use of fentanyl PCA with droperidol was associated with the lower incidence of PONV, compared with no use of PCA and the use of fentanyl PCA without droperidol; the use of fentanyl PCA without droperidol tended to be associated with the higher incidence of PONV, compared with no use of PCA; and the use of the reversal agent neostigmine 2 mg co-administrated with atropine 1 mg was associated with the lower incidence of PONV. On the other hand, age, BMI, the method of general anesthesia, or smoking habits were not associated with the incidence of PONV with either univariate or multivariate logistic regression analysis. The intraoperative use of prophylactic antiemetics and the postoperative use of rescue opioid analgesics other than PCA fentanyl were associated with the incidence of PONV with univariate logistic regression analysis, but not with the multivariate logistic regression analysis.

Reportedly, female sex, types of surgery (laparoscopic, gynecologic), the use of inhalation anesthesia versus TIVA, non-smoking status, longer duration of anesthesia, younger age (< 50 years), and the postoperative use of opioids are established risk factors of PONV^1)^. In the present study, however, age, non-smoking status, or the method of general anesthesia did not affect the incidence of PONV, possibly due to too small numbers of patients aged 50 years or more (*n* = 31 [2.5%]), smokers (*n* = 85 [7.0%]), and patients anesthetized with TIVA (*n* = 25 [2.0%]) to detect their significant effects. The postoperative use of opioids other than PCA fentanyl in 208 patients (17%) was associate with the incidence of PONV with the univariate logistic regression analysis, but not with the multivariate logistic regression analysis, possibly reflecting complex interactions among the use of opioids other than PCA fentanyl, and the use or no use of fentanyl PCA with or without droperidol. BMI was not associated with the incidence of PONV, in agreement with previous reports^1, 5)^.

Reportedly, prophylactic uses of antiemetics, such as dopamine receptor antagonists including droperidol and metoclopramide, corticosteroids including dexamethasone, and serotonin receptor antagonists including ondansetron and granisetron, are effective in reducing the incidence of PONV^1, 4)^. In the present study, however, the effect of prophylactic antiemetics given during anesthesia was detected with the univariate regression analysis, but not with the multivariate logistic regression analysis, possibly because prophylactic antiemetics were given only in much smaller number of patients (*n* = 136 patients [11.1%]), compared with our more recent study^4)^, and also because metoclopramide, which is less effective than droperidol^1)^, was more frequently used in the present study (metoclopramide in 96 patients [7.8%] versus droperidol in 40 patients [3.3%]). On the other hand, longer duration of anesthesia was associated with the increased incidence of PONV in accordance with the guideline^1)^. This finding could be related to the higher doses of potentially emetic drugs, such as inhalation anesthetics, administered during longer anesthesia.

One of major findings of the present study was that the use of fentanyl PCA with droperidol was associated with the significantly lower incidence of PONV, compared with no use of PCA and the use of fentanyl PCA without droperidol. This finding is in good agreement with previous studies showing that addition of droperidol to PCA infusate reduces the incidence of PONV^4, 6, 7)^. Conversely, the use of fentanyl PCA without droperidol tended to be associated with the higher incidence of PONV, compared with the no use of PCA. This finding agrees with the established finding showing that the postoperative use of opioids increases the incidence of PONV^1)^. Paradoxically, however, PONV occurred less frequently in patients receiving fentanyl PCA with droperidol than those not receiving fentanyl PCA. This finding might be explained by the fact that postoperative rescue opioids, which would increase the incidence of PONV^1)^, were given much more frequently in patients not receiving PCA (without co-administration of an antiemetic) than those receiving fentanyl PCA with an antiemetic droperidol (42.5% and versus 6.8%, *p* < 0.0001). Interestingly, not only PONV occurred less frequently but also postoperative rescue opioid analgesics were used less frequently in patients receiving fentanyl PCA with droperidol than those receiving fentanyl PCA without droperidol (6.8% versus 14.3%, *p* = 0.0003), possibly reflecting the opioid sparing effect of droperidol^8)^.

Another major finding of the present study was that the use of the reversal agent neostigmine 2 mg co-administrated with atropine 1 mg was associated with the lower incidence of PONV. A previous meta-analysis reported that neostigmine in doses ≥ 2.5 mg increases the incidence of PONV^9)^. However, a more recent meta-analysis revealed that the use of neostigmine combined with anticholinergic agents such as atropine did not increase the incidence of PONV^10)^. While previous studies thus have demonstrated that neostigmine increases or does not increase the risk of PONV^9, 10)^, no study has demonstrated that the use of a reversal agent neostigmine combined with anticholinergic agents decreases the incidence of PONV. Such discrepancies might result from the fact that all of previous studies investigating the effect of the reversal agent on PONV included only small numbers of patients (mostly < 100 patients)^9, 10)^, whereas the present study included a number of patients large enough to detect its effect (*n* = 1221). Although neostigmine is used to counteract residual neuromuscular block, co-administration of anticholinergics, such as atropine, are required because neostigmine given alone exerts cholinergic effects on the gastrointestinal tract (increased motility) and on the heart (bradycardia)^10)^. Reportedly, intrathecal neostigmine in doses ranging from 0.15 to 0.75 mg in human volunteers causes nausea and vomiting in a dose-dependent manner, probably via action on the brainstem^11)^, whereas intrathecal atropine in a small dose (0.1 mg) quite effectively prevents PONV induced by intrathecal morphine in patients undergoing cesarean section^12)^. Further, it is known that intravenous atropine can cross the blood-brain barrier to cause central effects^13)^. In addition, an anticholinergic agent scopolamine is well known to exert a potent antiemetic effect and is used to prevent PONV^1)^. In the present study, we used atropine 1 mg to prevent cholinergic effects of neostigmine 2 mg. Taken together, it seemed plausible that the antiemetic effect of atropine outweighed the emetic effect of neostigmine, thereby reducing the incidence of PONV in the present study, although a prospective large-scale study is required to verify our finding.

In the present study, we could learn historical changes in our anesthesia practice for LGS. During the study period (2007-2009), only a small number of patients were anesthetized with TIVA that could reduce the incidence of PONV, compared with inhalation anesthesia^1)^, whereas recently, all patients are anesthetized with TIVA^4)^. Ultrashort-acting remifentanil is used in all patients recently^4)^, while it was used less frequently (865/1221 [70.8%]) during the study period. After our previous study^4)^, prophylactic antiemetics using both droperidol and dexamethasone are given routinely, whereas prophylactic antiemetics, droperidol (*n* = 40) or less effective metoclopramide (*n* = 96)^1)^ were given only in 136 patients (11.1%) during the study period. Previously, the Japanese Ministry of Health, Labour, and Welfare approved the use of droperidol solely for sedation before and during anesthesia. However, it approved the off-label use of droperidol for treatment of PONV in September, 2009. Further, it approved the use of serotonin receptor antagonists, ondansetron and granisetron, for treatment of PONV in August, 2021, which would contribute to a further reduction of the incidence of PONV^1)^. After our previous study^4)^, a disposable PCA pump was exchanged from Baxter Infusor with a 30-min lockout time to a better adjustable disposable PCA pump with a 10-minute lockout time (COOPDECH^®^ PCA Syrinjector^®^; Daiken Medical, Osaka, Japan)^14)^. After the previous study^4)^, droperidol is routinely added to the PCA pump infusate, while the PCA infusate containing droperidol were used in only 384 patients (31.4%) during the study period. Multimodal analgesia was not used during the study period, whereas multimodal analgesia, e.g. employing rectus abdominis sheath block as well as transversus abdominis plane block^15)^, and intravenous acetaminophen^16)^, is now commonly used to reduce postoperative requirements of opioids and thus to reduce the incidence of PONV. During the study period, vecuronium was commonly used as a muscle relaxant, whereas recently, shorter-acting rocuronium is commonly used. At present, sugammadex for reversal of rocuronium-induced neuromuscular blockade is more commonly used than neostigmine because of the faster and more reliable reversal effect of sugammadex, although sugammadex may not reduce the incidence of PONV, compared with neostigmine^17)^.

This study had some limitations. First, the design of retrospective data analysis is clearly inferior to a prospective randomized and blinded investigation. A retrospective study design always has a substantial risk of bias. Therefore, we should be cautious in interpreting the data. Second, we could follow up the presence/absence of PONV only for nine hours after the return to the ward due to the follow-up period in the ward during the study period, which could underestimate the incidence of PONV, compared with a 24-hour follow-up time in our previous prospective study^4)^. Further studies are required to confirm the data shown in the present study.

In conclusion, longer duration of anesthesia was associated with the increased incidence of PONV. Addition of droperidol to the infusate of intravenous fentanyl PCA and the use of the reversal agent neostigmine co-administrated with atropine were effective in reducing the incidence of PONV, although further studies are required to validate our data. Additionally, we could learn historical changes in anesthesia practice for patients undergoing LGS at our institute.

## Funding

No funding was received.

## Author contributions

OK collected data. OK and MH analyzed data. OK wrote the first draft of the manuscript. MH edited the manuscript. All authors read and approved the final manuscript.

## Conflicts of interest statement

The authors declare that they have no conflict of interest associated with this manuscript.
